# On the Use of Cold Water in Cases of Old Ulcers

**Published:** 1801-07

**Authors:** Andrew Ferguson


### *On the Use of Cold Water in Cases of Old Ulcers.*

EIGHT years fince, being in a ftate of convalefence, I took a tour thirty miles up the country, to a place called Inchmarnoch, that I might have the benefit of a change of air, and a milk diet. This place being only a fhort diftance from a well, much frequented by difeafed perfons, called, from its fituation, Pananach Well, I had the curiofity to make frequent vifits to it, that I might mark its effects. It is fituated in a mountainous romantic country, and is very pleafant in the fummer feafon. The vallies are plentifully fupplied with fragrant birch, and the pure air defcending and mixing with their odours in every gentle breeze, owing to the loftinefs of the hills, renders the fcene particularly agreeable. The well is fituated upon the fide of a hill, and confifts of two fprings, which are very near each other, and yield a confiderable quantity of water, part of which is conveyed by a pipe into a large wooden trough, called a bath, placed in a houfe for the purpofe of immerfing the whole body; the remainder of the water runs down the hill in an irregular channel, which it has formed for itfelf. There are no buildings around the well to add to its appearance or convenience, except the fcenery of Nature, and a fmall houfe near the well which affords but little convenience to perfons who are difeafed. About an Englifh mile from the well there is a building called Pananach Lodge; this is fet apart for ladies and gentlemen, the poorer orders of patients having no accommodation allowed them. Thofe who cannot afford to refide at the Lodge, are under the neceffity of repairing to fome of the farm-houfes, two, three, or four miles diftant, fuch as Refantrach, Baletrach, Grey-ftone, and Dee-caftle; each of thefe generally accommodates convalefcents with lodgings and goat-milk through the fummer. During my ftay in this country, a number of difeafed perfons were attending the well, and applying the water; among whom there were four with old and foul ulcers; five with fcrophulous ulcers, affecting the joints and glands of their necks; one with ancylofis of the knee, accompanied with hectic fever; three with ftones in their urinary bladder; three with phthifis pulmonalis; one man with a large phagedenic ulcer upon his leg; and feveral with herpes upon various parts of their body. Thofe who were affected with ulcers, applied the water in the following manner: they took pieces of linen, and after dipping them in the water, and bathing their fores for five minutes or a quarter of an hour, laid the wet cloths upon the fores, and changed them as foon as they became dry. This was the general mode in which they used the water, but many of them bathed their whole body once or twice every day, and they all used a great quantity of the water internally. Thofe who were affected with ftones in the bladder, drank great quantities of the water, and ufed the exercife of walking afterwards. The diet of many of the poor patients is only oatmeal and water, which they boil together and call *pottage*. Milk is frequently fo fcarce, and was fo at that time, that few of them could procure it. Moft of the patients who were making trial of the water, had been difmiffed as incurable, either from hofpitals, or by individual practitioners, and had been advifed to make trial of the water. Many of the ulcers had a very bad appearance, and the cafes in general were of the worft kind. I could perceive in a very fhort time that the mode which they took of applying the wet cloths to their fores, was the very beft for giving the water a fair trial. This I was more convinced of, after I had feen its effects. Within a fortnight I could difcern a material alteration in the appearance of their fores; inftead of being foul, and having callous edges, their whole furface was of a redder colour, and full of new granulations of flefh. Several of the fcrophulous fores which were not accompanied with an affection of the bone, in a very fhort time had every appearance of healing. Even where there was an affection of the bone, the fores put on a more favourable appearance. There was a cafe of fcrophula, where the glands of the neck were much fwelled, with two large ulcers upon one fide, and one upon the other; in this cafe the wet cloths had a very quick effect in reducing the fize of the tumours, and cleanfing the furface of the ulcers. There were feveral of the cafes of fcrophulous ulcers combined with an affection of the bone; in thefe it could not be expected to be attended with much benefit, yet even here the fores looked better; the fetid fmell was much leffened, and the patients more vigorous. In the cafes of herpes it was alfo attended with advantage; it foon removed the crufts, and produced a more inflamed furface, which afterwards quickly healed. In a cafe of herpes upon the leg, and one upon the face and nofe, the application of the wet cloths proved a cure. In a cafe of herpes attended with fwelling of the leg it excited much pain and increafed the inflamation; the patient continued the ufe of the water, and after being fome months at home the herpes healed. The perfons afflicted with ftones remained much in the fame ftate except being better in health. I have been told, that in fome cafes the water has been known to bring away ftones; this may happen when they are fmall and capable of paffing through the ureters or urethra, but when they are larger it cannot have any effect in this this way. The quantity of water which fome of thefe patients drink is almoft incredible. If a ftone were obftructing the mouth of the bladder, by drinking too much water the pain would be increafed; this happened to be the cafe with one of the patients. It appears from the foregoing obfervations, that the Pananach water is attended with advantage as a topical application to old ulcers and herpes. In other complaints I cannot afcribe the benefit to it, but rather to the pure air of the country. It were worth while to enquire whether the advantage is derived from the nature of the water, the mode of its application, the change or purity of the air, or other circumftances. Patients who repair to wells of this kind place great confidence in the water, and ufe it with uncommon perfeverance. This contributes to keep the fores clean, and allows the ftimulus of the water to act more powerfully.—The Pananach water contains a large portion of iron, which is held in folution by the carbonic acid. The bottom and fides of the channel, which conveys the water from the well are covered with a yellow cruft (the creta ferrea of Fourcroy) which it depofits upon being expofed to the air. But to afcertain the prefence of iron with more precifion, I added to the water a little of the tincture of galls, which converted it into a high purple colour.—Thefe were fufficient evidences of its being a chalybeate water, and of courfe a very powerful tonic. When applied, therefore, to fores it muft have a very powerful action upon them. This was evident, for in the courfe of a few days I obferved an inflammation induced by the ftimulus of the water, and a new action in the morbid part. The water is always cold even in the warmeft day of fummer; this alfo contributes, by reducing the excefs of heat in the part, to add to the ftimulus which it poffeffes. The method in which the patients apply the water is well adapted for giving it full effect; and as they have nothing to do but take care of their fores, they are continually renewing the wet cloths, which keeps them fufficiently eafy, without the aid of ointment, and frees the fores of all the purulent matter. To afcertain whether any other water would be as effectual as the Pananach, I made application of common fpring water, and alfo of the Spa well at Aberdeen, which is a ftrong chalybeate water, in feveral cafes of old ulcers. The alteration in the appearance of the fores was nearly fimilar to what happened at Pananach, when the patient ufed the fame attention in applying it, and renewing the wet cloths. From this I conclude that all kinds of water poffeffing coldnefs, and a due proportion of carbonic acid, are beneficial in the cure of old ulcers, but more efpecially the chalybeate. It is not often that we can get patients to ufe any other water in the fame manner, or with the

ſame care and degree of confidence as they do the Pananach; and even the patients who have been there, relax in their attention as ſoon as they go home, and are, therefore, diſappointed of a cure. They erroneouſly imagine that no water will have the ſame effect, and leave off applying any thing to their ſores. Some of them, however, apply leaves of plantain, ivy, or ſuch like, which ſoon reduces them to their former degree of foulneſs. Were they to take my advice, I would order them to continue the application of cold water after the ſame manner as they did at Pananach, which would, from what I have witneſſed, be frequently attended with a complete cure. The pure air at Pananach is certainly very ſerviceable in many caſes, and frequently produces a more vigorous ſtate of the ſolids in general, eſpecially when it is accompanied with a nouriſhing diet. This is what a number of thoſe who go to Pananach cannot procure; yet in general they appear more vigorous even with what ſuſtenance it affords. When the body is invigorated by whatever means, it muſt produce an alteration upon the ſtate or condition of the ulcerated ſurface. This is to be conſidered, therefore, when we are comparing the advantages derived from this and the water in cities, where patients have not the opportunities of breathing ſuch pure air. Therefore, when people can get to a part where they can breathe a pure air, and make uſe of chalybeate or medicinal water of any kind at the ſame time, they will have a greater chance of cure. The ſummer ſeaſon is chiefly the time for uſing the water to moſt advantage at Pananach. In the winter the country is ſo cold and barren that none attend it. Patients with ulcers can, however, in almoſt all parts, procure good ſpring water, which they may ſubſtitute for it with advantage; and in Aberdeen they have a chalybeate ſpring which is in no reſpects inferior to the Pananach, except in its ſituation. From what I have ſeen of the good effects of wet cloths applied out of cold water to old ulcers, I am led to conſider it as one of the moſt powerful and effectual agents which can be uſed, or has yet been diſcovered. I would recommend a faithful trial of it in every caſe deemed incurable. I would adviſe the patients to perſevere in uſing it a ſufficient length of time, otherwiſe no cure is to be expected. The length of time requiſite for its ſucceſs is always proportioned to the virulence of the ſores, their number, ſize, and nature.

ANDREW FERGUSON.

